# Advection–Dispersion Behavior for Simulation of H-3 and Pu-238 Transport in Undisturbed Argillaceous Shale of a Near-Surface Repository

**DOI:** 10.3390/toxics11020124

**Published:** 2023-01-27

**Authors:** Yunfeng Shi, Song Yang, Enhui Wu, Longjiang Wang, Wenjie Chen, Weijia Xiong, Yanna Zhang, Aiming Zhang, Bing Lian

**Affiliations:** 1 Department of Nuclear Environmental Science, China Institute for Radiation Protection (CIRP), Taiyuan 030006, China; 2 School of Nuclear Science and Engineering, East China University of Technology, Nanchang 330013, China; 3 CNNC Environmental Protection Corporation (ECPC), Beijing 100045, China

**Keywords:** Pu-238, H-3, undisturbed column experiment, batch experiment, stream tube model

## Abstract

In this study, a column experiment was employed to evaluate the nuclide migration behavior in the surrounding rock medium of a near-surface disposal site in China and to investigate the advection–dispersion behavior of tritium (H-3) and plutonium-238 (Pu-238) in highly weathered argillaceous shale. A reasonable numerical model was selected to fit the experimental breakthrough curves (BTCs) and to obtain the relevant migration parameters. The results show the following: (1) the internal structure of the highly weathered argillaceous shale exhibited heterogeneity, and the nuclide migration BTC showed characteristics of a “curve peak moving forward” and a “tail curve trailing”; (2) compared with other models, the stream tube mode could better fit the BTCs and obtain the average dispersion coefficient <*D*>, average distribution coefficient <*K_d_*>, and other parameters; (3) compared to the results of the batch experiment, the distribution coefficient *K_d_* obtained from the column experiment was smaller than that obtained from the batch experiment, which is speculated to be due to the influence of contact time and the contact area between the nuclide and the medium.

## 1. Introduction

Radioactive waste can be classified as high-level, intermediate-level, and low-level wastes. Currently, the international common disposal method for intermediate-level and low-level wastes is to use near-surface treatment in order to ensure that there are no adverse impacts on biological health within the design lifetime [1]. In the Safety Case concept proposed by the International Atomic Energy Agency (IAEA), near-surface repositories require further research to understand the migration behavior of radionuclides in the surrounding rock in order to determine the key migration parameters [2,3,4]. Argillaceous shale is a rock type that is widely distributed in China. It is the main wall rock of a near-surface disposal site in China because of its strong adsorption capacity for nuclides and water swelling property [5,6].

Pu (atomic number 94) is mainly available in the forms of Pu-238, Pu-239, and Pu-240 nuclides in low-level and intermediate-level radioactive wastes, in which Pu-238 (T_1/2_ = 8.77 × 10^1^ years), mainly from tests of relevant nuclear weapons, exhibits a long life, high activity, and high toxicity, and it is the key nuclide in low- and intermediate-level radioactive wastes [7,8]. There have been many studies on the adsorption and diffusion behaviors of Pu in different media [9]. For example, Xie studied the adsorption dynamic behavior of Pu-239 on sand, highly weathered granite, and silty clay using a batch experiment [10]. Dang studied the diffusion behavior of Pu in granite through a penetration diffusion experiment and discussed the influences of the chemical forms of Pu on the diffusion behavior [11]. In terms of convection dispersion migration behavior, although a lot of studies have reported the convection dispersion behavior of Pu in different media [12,13,14], most of the media are porous filling media, and there is limited research on the migration behavior of Pu in undisturbed fractured media in the field.

Column experiments can be used to study the advection–dispersion migration behavior of solutes in various solid media [15,16]. According to the type of solid media used, column experiments can be divided into packed column and undisturbed column experiments. Most studies have utilized the undisturbed column experiment, and it has always been favored by scholars because it better reflects the actual situation of the field formation medium. However, it has disadvantages, such as difficulties in obtaining and storing undisturbed media and in eliminating the column wall effect [17,18,19].

The numerical models that can be used to simulate fractured media include the equivalent porous medium model, the dual porosity/feasibility model, the stream tube model, the discrete frame network model, and the hybrid approach model [20]. The equivalent porous medium and dual porosity/feasibility models are widely used for indoor small-scale column experiments. The internal structure is mostly homogeneous for columns with highly weathered fractured media (or columns filled with fractured media), and a good fitting effect can be achieved when using an equivalent porous media model [21,22]. The weathering degree of fractured media is weak, the internal structure is highly heterogeneous, and the solute breakthrough curves (BTCs) have a clear “preferential flow” phenomenon. Using a dual porosity/feasibility model for such a medium can have a good fitting effect [23,24]. However, for highly weathered fractured media, due to the strong heterogeneity of the internal structure and the obvious tailing phenomenon of the BTCs, research on a numerical model that can be used to better fit the penetration curve is rarely reported.

As mentioned above, at present, the following problems exist when attempting to determine the migration behavior of radionuclides in groundwater environments: (1) in column experiments, the columns are mostly filled with media, and in undisturbed medium experiments, the columns are filled with too little media, and (2) in terms of numerical models, although a lot of numerical models have been established, there are few numerical simulation experiments of strongly weathered fractures. Therefore, in this study, batch and column experiments were used to study the adsorption behavior and convection dispersion migration behavior of H-3 and Pu-238 in the surrounding rock medium of a near-surface repository in China. Different numerical models (the equivalent porous medium model, the two-region nonequilibrium transport model, and the stream tube model) were fitted to the solute penetration curve (BTC), and the key migration parameters were determined. The research results can provide technical support for studying the radionuclide adsorption retardation performance of the surrounding rock medium of low- and intermediate-level solid waste disposal sites.

## 2. Theory of Advection and Dispersion

### 2.1. Equivalent Porous Medium Model

The equivalent porous medium model is a numerical model that equates the fractured medium to the porous medium. It can be applied to the numerical simulation calculation of water bodies and solute transport behavior in homogeneous water passages generated by strongly weathered fractures. Its one-dimensional equation is as follows:

(1) $$\frac{\partial }{\partial t}\left(\theta c_{r}+\rho_{b}s\right)=\frac{\partial }{\partial x}\left(\theta D\frac{\partial c_{r}}{\partial x}-J_{w}c\right)-\theta \mu_{l}c_{r}-\rho_{b}\mu_{s}s+\theta r_{l}\left(x\right)+\rho_{b}r_{s}\left(x\right)$$

where $c_{r}$ is the volume-averaged or resident concentration of the liquid phase (ML^−3^); s is the concentration of the adsorbed phase (MM^−1^); D is the dispersion coefficient (L^2^T^−1^); θ is the volumetric water content (L^3^L^−3^); $J_{w}$ is the volumetric water flux density (LT^−1^); $\rho_{b}$ is the soil bulk density (ML^−3^); $\mu_{l}$ and $\mu_{s}$ are the first-order decay coefficients for the degradation of the solute in the liquid and adsorbed phases, respectively (T^−1^); $r_{l}$ (ML^−3^T^-1^) and $r_{s}$ (MM^−1^T^−1^) are the zero-order production terms for the liquid and adsorbed phases, respectively; x is the distance (L); and t is the time (T).

The solute adsorption by the solid phase is expressed using a linear isotherm as follows:

(2) $$s=K_{d}c_{r}$$

where $K_{d}$ is an empirical distribution constant (M^−1^L^3^). Using (2) and assuming a steady-state flow in a homogeneous soil, (1) may be rewritten as follows:

(3) $$R\frac{\partial c_{r}}{\partial t}=D\frac{\partial^{2}c_{r}}{\partial x^{2}}-v\frac{\partial c_{r}}{\partial x}-\mu c_{r}+r\left(x\right)$$

where v ($v=\frac{J_{w}}{\theta }$) is the average pore-water velocity; *R* is the retardation factor given by $R=1+\frac{\rho_{b}K_{d}}{\theta }$; and μ and r are combined first- and zero-order rate coefficients: $\mu =\mu_{l}+\frac{\rho_{b}K_{d}\mu_{s}}{\theta }$, $r\left(x\right)=r_{l}\left(x\right)+\frac{\rho_{br_{s}\left(x\right)}}{\theta }$, respectively.

### 2.2. Two-Region Nonequilibrium Model

The two-region nonequilibrium model divides the fractured media into mobile (flowing) and immobile (stagnant) regions according to permeability. The model has a good fitting effect for the “preferential flow” generated by large fractures and the tailing phenomenon of the solute BTCs due to the existence of stagnant regions. The two-region solute transport model is given by

(4) $$\begin{array}{c} \left(\theta_{m}+f\rho_{b}K_{d}\right)\frac{\partial c_{m}}{\partial t}=\theta_{m}D_{m}\frac{\partial^{2}c_{m}}{\partial x^{2}}-J_{w}\frac{\partial c_{m}}{\partial x}-a\left(c_{m}-c_{im}\right)-\\ \left(\theta_{m}\mu_{l,m}+f\rho_{b}K_{d}\mu_{s,m}\right)c_{m}+\theta_{m}\gamma_{l,m}\left(x\right)+f\rho_{b}\gamma_{s,m}\left(x\right) \end{array}$$

(5) $$\begin{array}{c} \left(\theta_{m}+\left(1-f\right)\rho_{b}K_{d}\right)\frac{\partial c_{im}}{\partial t}=a\left(c_{m}-c_{im}\right)-\\ \left(\theta_{im}\mu_{l,m}+\left(1-f\right)\rho_{b}K_{d}\mu_{s,im}\right)c_{im}+\theta_{im}\gamma_{l,m}\left(x\right)+\left(1-f\right)\rho_{b}\gamma_{s,im}\left(x\right) \end{array}$$

where the subscripts m and im refer to the mobile and immobile liquid regions, respectively; $J_{w}=v\theta =v_{m}\theta_{m}$ is the volumetric water flux density (LT^−1^); f represents the fraction of adsorption sites that equilibrates with the mobile liquid phase; and a is a first-order mass transfer coefficient (T^−1^) governing the rate of solute exchange between the mobile and immobile liquid regions. Note that $\theta =\theta_{m}+\theta_{im}$.

### 2.3. Stream Tube Model

The simulation medium is regarded as a series of mutually independent vertical soil columns. The flow in these columns is regarded as a pipe flow, and the equilibrium model describes the flow process in each pipe. However, the migration parameters in each pipe flow are randomly distributed. The pairs of stochastic parameters in the stream tube model for transport in each stream tube are obtained from a bivariate lognormal joint probability density function. Because of their relatively low coefficient of variation, the same values for θ and $\rho_{b}$ are used for each stream tube. The joint probability density functions of v, in conjunction with D, K_d_, and a, are written as f(v,D), f(v,K_d_), and f(v,a), respectively. The general bivariate lognormal joint pdf is defined as follows:

(6) $$f\left(v,\eta \right)=\frac{1}{2\pi \sigma_{v}\sigma_{\eta }v\eta \sqrt{1-\rho_{v\eta }^{2}}}exp\left(-\frac{Y_{v}^{2}-2\rho_{v\eta }Y_{v}Y_{\eta }+Y_{\eta }^{2}}{2\left(1-\rho_{v\eta }^{2}\right)}\right)\;\;\;\;\;\;\;\;\;\;\;\;\;\;\;\;\;\;\;\;\;\;\;\;\;\;\;\;\;$$

(7) $$Y_{v}=\frac{ln\left(v\right)-\mu_{v}}{\sigma_{v}}\;\;\;\;\;\;\;\;\;\;\;\;\;\;\;\;\;\;\;\;\;\;\;\;\;\;\;\;\;\;\;\;\;\;\;\;\;\;\;\;\;\;\;\;\;\;\;\;\;\;\;\;\;\;\;\;\;\;\;\;\;\;\;\;\;\;$$

(8) $$Y_{\eta }=\frac{ln\left(\eta \right)-\mu_{\eta }}{\sigma_{\eta }}\;\;\;\;\;\;\;\;\;\;\;\;\;\;\;\;\;\;\;\;\;\;\;\;\;\;\;\;\;\;\;\;\;\;\;\;\;\;\;\;\;\;\;\;\;\;\;\;\;\;\;\;\;\;\;\;\;\;\;\;\;\;\;\;\;\;$$

(9) $$\rho_{v\eta }=\langle Y_{v}Y_{\eta }\rangle =\int_{0}^{\infty }\int_{0}^{\infty }Y_{v}Y_{\eta }f\left(v,\eta \right)dvd\eta$$

where η denotes D, $K_{d}$, or a; μ and σ are the mean and standard deviation of the log-transformed variable, respectively; and $\rho_{v\eta }$ is the correlation coefficient between $Y_{v}$ and $Y_{\eta }$.

## 3. Experiment

### 3.1. Experimental Device

**Figure 1:**
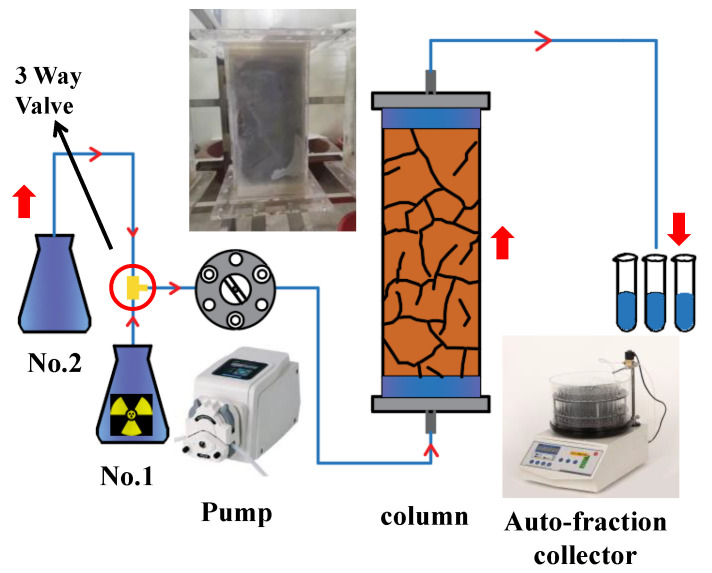
A reliable ADE device with a glass column apparatus in this study.

The experimental apparatus shown in Figure 1 was used for the dynamic flow column system. The device mainly includes a high-performance glass cylinder (modified type, Shanghai SuKe Industrial Co., Ltd., Shanghai, China); a high-performance and precise isocratic flow pump (MASTERFLEX L/S, Cole-Parmer Instrument Co., Barrington, IL, USA); an auto-fractional collector (BXZ-100, Shanghai Huxi Industrial Co., Ltd., Shanghai, China); and two reservoirs that contained groundwater (GW), a radiotracer (H-3/Pu-238), and the associated Teflon^®^ (PTFE) tubes and port connectors. The glass geometry is listed in Table 1, and its cuboid consisted of a pressure-resistant glass (<1 MPa) with a length of 360 mm, a width of 120 mm, and a height of 120 mm. It was filled with 12.39 ± 0.50 g of undisturbed rock in a bulk density of 2.39 ± 0.05 g/cm^3^_._ A pressure sensor and switch drain valves were equipped within the flowing column system, providing a constant and stable liquid flow rate.

### 3.2. Field Sample

**Figure 2:**
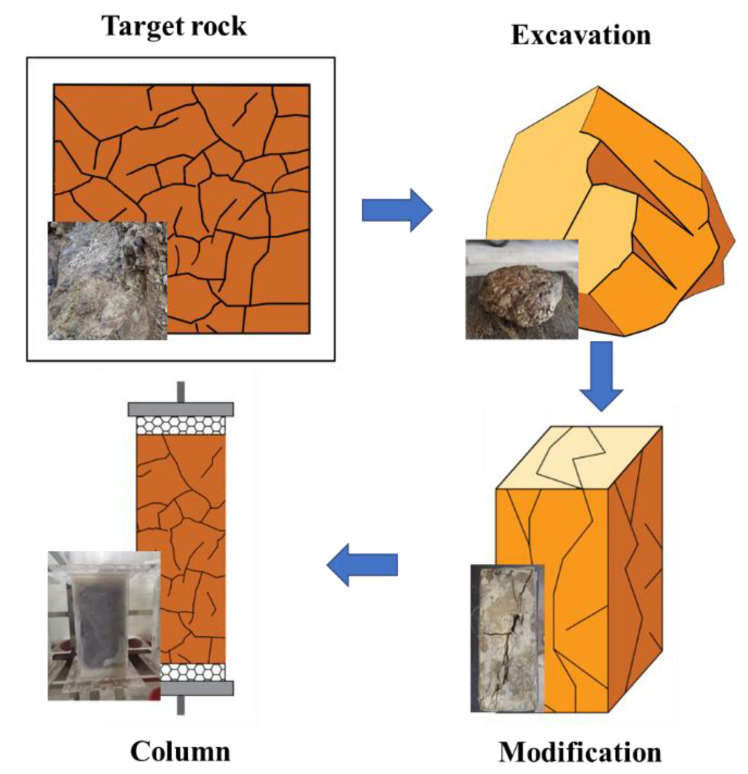
Collection process of shale samples.

The highly weathered argillaceous shale and groundwater samples were collected from a near-surface repository in southern China. The “freezing and grinding” method was used to ensure the collection of undisturbed, highly weathered argillaceous shale samples. After the fragile argillaceous shale was frozen and fixed by liquid nitrogen, it was polished into a cube using an angle grinder and other equipment. The sampling process is shown in Figure 2.

### 3.3. Analysis of Argillaceous Shale and Groundwater

An X-ray diffraction spectrometer (D/max-2500PC), a wavelength-dispersive X-ray fluorescence spectrometer (Axios-mAX), and an ultraviolet–visible spectrophotometer (UV-1100) were used to analyze the mineral composition, chemical composition, organic matter, and cation exchange capacity of the rock samples. The test results are shown in Table 2 and Table 3. In addition, a conductivity meter (FE38), an ion chromatograph (ICS-1100), an automatic potentiometric titrator (AT-510), and a plasma mass spectrometer (ELEMENT XR) were used to analyze the chemical composition of the groundwater. The test results are shown in Table 4.

### 3.4. Batch Sorption Test

The experiment was used to test the adsorption capacity of the highly weathered argillaceous shale on Pu-238 and to determine the distribution coefficient as follows:

(10) $$K_{d}=\frac{Concentration\;of\;nuclides\;per\;unit\;mass\;of\;soild\;medium\;(Bq/kg)}{Concentration\;of\;nuclides\;per\;unit\;volume\;of\;water\;body\;\left(Bq/L\right)}$$

First, the shale was crushed and sieved to achieve a particle size below 75 μm. Then, 1 g of crushed shale was added to 9 mL of groundwater into a 15 mL centrifuge tube, and 1 mL of Pu-238 solution (2000 Bq) was added. After shaking and mixing for 7 days, the test tube was centrifuged for 20 min at 800 r/min in a centrifuge, and the supernatant was extracted to measure the content of Pu-238. The calculated distribution coefficient of the Pu-238 on the strongly weathered argillaceous shale was equal to 2170 L/kg. In addition, the pH = 3.50 ± 0.20 and Eh = 0.24 ± 0.02 V of the liquid sample were measured after the experiment, and Pu(III) was the chemical form according to the pH–Eh diagram [25].

### 3.5. Water Saturation

A peristaltic pump was opened, and the valve was adjusted to pass the groundwater into the column from the bottom at a flow rate of 3 mL/min in order to ensure that all the pores in the whole column were filled with groundwater. The concentrations of Na, Mg, Ca, and K in the effluent were measured using inductively coupled plasma–optical emission spectrometry (ICP–OES, iCAP 7000, Thermo, Waltham, MA, USA) at various concentrations within 5% of the corresponding liquid-phase concentrations, and it was determined that water saturation had been reached.

### 3.6. Nonreactive Tests—H-3

The migration behavior of radionuclides can be affected by the structure of the water passage and the chemical reaction simultaneously. To determine the structure of the water passage in the medium, a non-reactive tracer (H-3) was used to perform a migration behavior experiment under advection–dispersion conditions. H-3 (500 Bq/mL) was placed into container 1, and the underground water sample was placed into container 2. The peristaltic pump was opened to inject groundwater into the column at a flow rate of 25 mL/min. After the outflow was stabilized, the valve was adjusted to inject the H-3 solution 1000 Bq (2 mL), and then the valve was quickly adjusted to continue injecting the groundwater. The effluent was collected at the outlet every 1 min, and a liquid scintillator analyzer (LSA3000, Shanghai SIM-MAX Technology Co., Ltd, Shanghai, China) was used to analyze the H-3 and Pu-238 activities in the test solution with a scintillation cocktail. The experiment was concluded when the H-3 content in the effluent was lower than the lower detection limit, and a BTC was obtained.

### 3.7. Reactive Tests—Pu-238

After the H-3 solution in container 1 was replaced with Pu-238 (2000 Bq/mL), Pu-238 was injected into the column via an instantaneous injection according to the same experimental process. The groundwater flow was maintained at 25 mL/min, and it was continuously injected into the argillaceous shale. When the outflow was stable, the valve was adjusted to inject a 4000 Bq (2 mL) Pu-238 solution, and then it quickly adjusted to continue injecting the groundwater. The outflow was collected at the outflow outlet every 1 min. A liquid scintillator was used to analyze and test the activity of Pu-238 in the solution. The experiment was concluded when the content of Pu-238 in the outflow was lower than the lower limit of detection, and a BTC was obtained.

### 3.8. Mathematical Model and Parameter Estimations

STudio of ANalytical MODels (STANMOD) is a model package containing several analytical models that solve the solute transport behavior in porous media based on the advection–dispersion equation. It includes a deterministic model and a stochastic model. The deterministic model includes the deterministic equilibrium model and the deterministic nonequilibrium model (the two-site nonequilibrium model and the two-region nonequilibrium model). A stochastic model is a stream tube model.

To determine the fitting effect, the software uses the root mean square error (RMSE), and it is expressed as follows:

(11) $$RMSE=\sqrt{\frac{\sum_{i}^{N}{\left(C^{p}-C^{e}\right)}^{2}}{N}}$$

where $C^{p}$ denotes the results of the numerical fitting, and $C^{e}$ denotes the experimental data.

## 4. Results and Discussion

### 4.1. Breakthrough Curves of H-3

**Figure 3:**
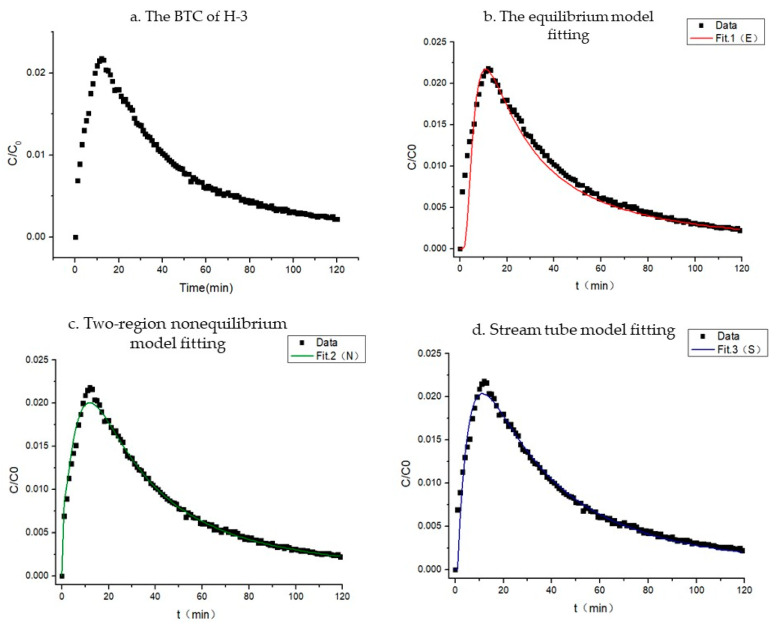
The experimental breakthroughs of H-3 and fitting curves.

A column experiment was used to obtain the BTC of the advection–dispersion transport of H-3 in highly weathered argillaceous shale. As shown in Figure 3a, the BTC of H-3 showed the phenomena of “peak moving forward” and “tail dragging”, which indicates that the water flow resulted in a “preferential flow” inside the column. The equilibrium model, the nonequilibrium model (two-region nonequilibrium model), and the stream tube model were used to fit the BTC of H-3. The fitting effect is shown in Figure 3b–d and Table 5. The equilibrium model (RMSE = 0.930), the two-region nonequilibrium model (RMSE = 0.987), and the stream tube model (RMSE = 0.996) well-reflected the characteristics of the curve. However, the analysis parameters indicated that the dispersion coefficient (D = 19.81 cm^2^/min) obtained from the equilibrium model and the dispersion coefficient (D = 17.5 cm^2^/min) obtained from the two-region nonequilibrium model were significantly larger, which is inconsistent with the actual situation. Therefore, the stream tube model can better fit the advection–dispersion behavior of H-3 in highly weathered argillaceous shale.

### 4.2. Breakthrough Curves of Pu-238 

**Figure 4:**
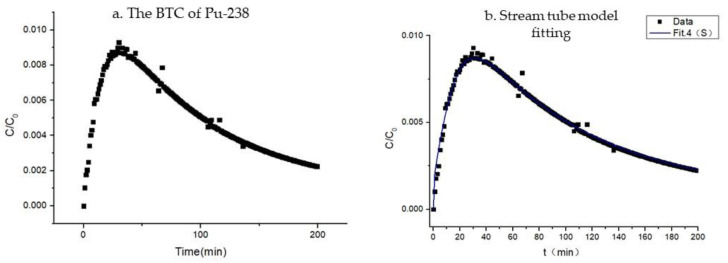
The experimental breakthroughs of Pu-238 and fitting curves.

The BTC of Pu-238 also exhibited the phenomena of “peak moving forward” and “tail dragging”. The stream tube model was used to fit the advection–dispersion behavior of Pu-238 in the highly weathered argillaceous shale, and the fitting results are shown in Figure 4 and Table 5. The fitting results of the H-3 ‘BTC were inputted as fixed values into the model, and other parameters were fitted to obtain the ensemble average distribution coefficient <K_d_> = 46.9 L/kg and the correlation coefficient between the flow rate and the distribution coefficient $\rho_{\mathrm{Kd}\;*\;V}$ = −1, indicating that the flow rate was negatively correlated with the distribution coefficient. In addition, the pH = 3.50 ± 0.20 and Eh = 0.24 ± 0.02 V of the liquid sample were measured after the experiment, and Pu(III) was the chemical form according to the pH–Eh diagram.

### 4.3. Parameter Comparison

The chemical species of Pu is complex, and the chemical species affects the adsorption mechanism of Pu. Therefore, it was necessary to analyze the chemical species of Pu-238 when comparing the distribution coefficients obtained from the batch experiments and the column experiments. According to the measured values of the pH and the Eh of the liquid after the batch and column experiments and the thermodynamic database [25], the chemical species of Pu-238 in the batch and column experiments was determined to be Pu(III). Therefore, the influence of the chemical form of plutonium was not considered when comparing the distribution coefficients obtained from the batch experiments and column experiments.

A comparison of the distribution coefficient of Pu-238 obtained in the batch experiment (*K_d_* = 2170 L/kg) indicated that the distribution coefficient obtained in the column experiment (<*K_d_*> = 46.9 L/kg) was small. The main reason for the difference in the distribution coefficient between the two experiments is the way of sample treatment. Because the argillaceous shale was broken in the batch experiment and the specific surface area of the argillaceous shale was artificially increased, the contact area between the nuclide and the argillaceous shale was larger than that in the column experiment, and Pu-238 could be better adsorbed on the argillaceous shale, so the distribution coefficient obtained in the batch experiment was larger than that obtained in the column experiment. In addition, in order to ensure that the argillaceous shale and the Pu-238 could reach adsorption equilibrium in the batch experiment, the contact time between the argillaceous shale and Pu-238 was extended. The migration of the nuclides in the column experiment was completed under the condition of maintaining a certain flow rate. Therefore, the contact time between the argillaceous shale and Pu-238 was shorter than that in the batch experiment, which also led to the distribution coefficient obtained in the batch experiment being greater than that obtained in the column experiment.

## 5. Conclusions

This study investigated the advection–dispersion behavior of H-3 and Pu-238 in the fractures of highly weathered argillaceous shale surrounding the rock medium of a near-surface disposal site in China. According to the fitting results of the BTC, an appropriate mathematical model was selected, and the key migration parameters were obtained. The research results are as follows:

1. The advection–dispersion behavior of H-3 and Pu-238 in the highly weathered argillaceous shale showed “preferential flow”, reflecting the strong heterogeneity of highly weathered argillaceous shale.
2. The equilibrium model, the nonequilibrium model (in the two-region mode), and the stream tube model were used to fit the BTC. The results show that the stream tube model can better fit the curve characteristics, indicating that the water-carrying capacity of each channel of media varies greatly and is complex.
3. A comparison of the distribution coefficients obtained from the column and batch experiments indicated that the distribution coefficient obtained from the column experiment was smaller, and an analysis was necessary mainly due to the contact area and contact time.

## Figures and Tables

**Table 1:** The experimental glass filled and compacted with crushed granite.

Item	Location	Length × Width × Height (cm)	Weight (g)	Density (g/cm^3^)	Porosity
Argillaceous shale	Near-surface repository in China	36 × 12 × 12	12.39 ± 0.50	2.39 ± 0.05	0.18 ± 0.03

**Table 2:** Analysis results of argillaceous shale.

Item	SiO_2_ (%)	Al_2_O_3_ (%)	Fe_2_O_3_ (%)	MgO (%)	CaO (%)	Na_2_O (%)
Argillaceous shale	60.02	17.50	6.89	2.06	0.516	0.740
	K_2_O (%)	MnO (%)	TiO_2_ (%)	P_2_O_5_ (%)	Loss on ignition (%)	FeO (%)
	3.90	0.09	0.699	0.111	7.42	1.11
	Sr (μg/L)	Ni (μg/L)	Pu (ppm)	Organic matter (g/kg)	Cation exchange capacity (cmol/kg)	
	85.3	41.6	<1	22.1	21.5	

**Table 3:** Argillaceous shale mineral (%).

Item	Dolomite	Calcite	Quartz	Plagioclase	Potash Feldspar
Argillaceous shale	8	-	28	5	-
	Illite	Amphibole	Chlorite	Pyrite	Montmorillonite
	31	-	2	-	26

**Table 4:** Analysis results of groundwater.

Item	F^−^	Cl^−^	NO_3_^−^	SO_4_^2−^	Na^+^	K^+^	Mg^2+^	Ca^2+^
Groundwater	(mg/L)							
	0.487	1.82	2.23	42.9	24.3	14.5	2.89	19.1
	Sr	Ni	Pu	Conductivity	pH	Turbidity (UNT)		
	μg/L		ppm	μS/cm				
	257	1.1	<1	271	8.19	0.43		

**Table 5:** The parameters for fitting BTCs.

NO	Model	*v* (cm/min)	*D* (cm^2^/min)	*K_d_* (L/kg)	*β* $\;(\frac{\theta_{m}}{\theta })$		*a*		RMSE
FIT1 (H-3)	Equilibrium	0.026	19.812	0	-		-		0.930
FIT2 (H-3)	Two-region non-equilibrium	0.426	17.514	0	0.191		0.832		0.987
NO	model	<*v*> (cm/min)	<*D*> (cm^2^/min)	<*K_d_*> (L/kg)	*σ_V_*	*σ_D_*	*σ_Kd_*	$\rho_{Kd*V}$	RMSE
FIT3 (H-3)	Stream tube	0.490	0.292	0	1.111	3.120	0		0.996
FIT4 (^238^Pu)	Stream tube	0.490	0.292	46.924	1.111	3.120	38.512	−1	0.957

## Data Availability

Not applicable.
